# *Leishmania* Spp-Host Interaction: There Is Always an Onset, but Is There an End?

**DOI:** 10.3389/fcimb.2019.00330

**Published:** 2019-09-19

**Authors:** Fatima Conceição-Silva, Fernanda N. Morgado

**Affiliations:** ^1^Laboratory of Immunoparasitology, Oswaldo Cruz Institute, IOC/Fiocruz, Rio de Janeiro, Brazil; ^2^Laboratory of Leishmaniasis Research, Oswaldo Cruz Institute, IOC/Fiocruz, Rio de Janeiro, Brazil

**Keywords:** leishmaniasis, parasite evasion mechanisms, immune response, parasite-host interaction, parasite persistence

## Abstract

For a long time Leishmaniasis had been considered as a neglected tropical disease. Recently, it has become a priority in public health all over the world for different aspects such as geographic spread, number of population living at risk of infection as well as the potential lethality and/or the development of disfiguring lesions in the, respectively, visceral and tegumentary forms of the disease. As a result, several groups have been bending over this issue and many valuable data have been published. Nevertheless, parasite-host interactions are still not fully known and, consequently, we do not entirely understand the infection dynamics and parasite persistence. This knowledge may point targets for modulation or blockage, being very useful in the development of measures to interfere in the course of infection/ disease and to minimize the risks and morbidity. In the present review we will discuss some aspects of the *Leishmania* spp—mammalian host interaction in the onset of infection and after the clinical cure of the lesions. We will also examine the information already available concerning the parasite strategy to evade immune response mainly at the beginning of the infection, as well as during the parasite persistence. This knowledge can improve the conditions of treatment, follow-up and cure control of patients, minimizing the potential damages this protozoosis can cause to infected individuals.

## Leishmaniasis: A Brief Introduction

Leishmaniasis is an infectious disease caused by parasites of the genus *Leishmania* that affect humans and other animals. They are transmitted by insects popularly known as sand flies (Order Diptera, family *Psychodidae*, subfamily Phlebotominae) and can cause different clinical presentations, from cutaneous, and/or mucosal lesions (tegumentary leishmaniasis) up to visceral infection with tropism by the lymphohematopoietic system (visceral leishmaniasis). Due to its potential for lethality, mainly in visceral forms, as well as its dispersion in several continents with large population groups under infection risk, it is considered by WHO as one of the priority attention diseases (World Health Organization, 2010, 2017; Alvar et al., 2012).

The resolution WHA60.13, published during the World Health Assembly in 2007, aims to develop different actions all over the world in order to promote leishmaniasis control both by improving diagnosis and treatment access and by establishing or fortifying national control programs (World Wealth Assembly, 2007). Nowadays, leishmaniasis is described in 98 countries and several others are under risk of infection due to climate changes and/or population mobility (by migration and travel) (Ait Kbaich et al., 2017; Azimi et al., 2017; Baylis, 2017; World Health Organization, 2017, among others). In this sense, several diagnostic possibilities have emerged in recent years, and the serological test rK39 is among the most promising rapid tests due to its high sensitivity and specificity for the diagnosis of visceral leishmaniasis in symptomatic and asymptomatic patients (Maia et al., 2012; Boelaert et al., 2014; Bangert et al., 2018).

In order to develop new drugs and less invasive diagnostic procedure, it is necessary to better understand the parasite-host interaction and the factors that can promote disease severity and control. Several factors have been implicated in determining, or at least facilitating, the development of leishmaniasis in humans (reviewed by Conceição-Silva et al., 2018), and the consensus indicates that the presence of clinically detectable leishmaniasis occurs due to multiple factors related to both parasite and host. In this connection, in the last few years, an increased number of evidences have pointed several mechanisms that can take place according to the necessity for survival from both sides.

At the host side, the presence of malnutrition, comorbidities, extremes of age, amongst others may facilitate the evolution of leishmaniasis to more severe forms, since many of these factors act direct or indirectly on the ability of the immune system to respond adequately to the presence of the parasite (reviewed by Conceição-Silva et al., 2018). For example, in HIV-*Leishmania* coinfection patients, VL quickly accelerates the onset of AIDS and shortens their life-expectancy. Also, HIV increases the risk of clinical VL and the risk of atypical leishmaniasis (Freitas-Junior et al., 2012; Távora et al., 2015; Henn et al., 2018). In addition, during thousands of years, parasites and humans have co-evolved and, consequently, their surviving systems have been structured to avoid danger and damage. As a result, parasites have developed intricate strategies to subvert, modify and/or inhibit the host immune response. Positive selection pressure has improved species survival into the host environment based on more adapted individuals. Thus, the “good” parasite is that one capable of surviving while causing minimal damage to its host. The result of this encounter may lead to equilibrium (parasitic persistence) or disequilibrium (disease) of the parasite-host relationship. This point is easily demonstrated by the clinical disease onset in individuals under immunosuppressive status (recently reviewed by Akuffo et al., 2018; Conceição-Silva et al., 2018) even with no previous story of infection.

Two natural events proving the persistence of parasite are represented by 1- mucosal lesions produced years after primary cutaneous lesions (Leite et al., 2012; Ávila et al., 2018), and 2- the Post-Kal-azar dermal leishmaniasis (PKDL) after VL recovering (Ganguly et al., 2010; Singh et al., 2012; Zijlstra, 2016). In both cases, the same *Leishmania* species is able to produce, after healing, a different clinical form from primary manifestations. Although these manifestations appear in part of patients, since mainly *Leishmania donovani* is responsible for PKDL, and just few *Leishmania* species can produce mucosal forms, the importance of parasite in these late clinical manifestations is evident. Furthermore, isolates obtained from PKDL patients genetically differ from strains of patients who present just VL (Mishra et al., 2013). These differences may play a role in the pathogenesis of PKDL and in drug resistance to sodium antimony gluconate, miltefosine, and paromomycin (Mishra et al., 2013).

At the parasite side, different pathways to avoid or subvert the immune response have been demonstrated (reviewed by Duque and Descoteaux, 2015; Mandell and Beverley, 2016; Conceição-Silva et al., 2018, among others). The mechanisms of escape of *Leishmania* spp in their hosts is so varied that we decided to focus on some aspects of the interaction, notably those strategies observed at the beginning of infection, escape from NETs, cleavage of molecules from complement system, strategies to entry, and to survive inside permissive cells and subversion of macrophages and lymphocytes, as well as the capacity to use the normal regulatory immunological mechanisms to replicate and to establish infection.

One of the first steps of the *Leishmania*-host interaction occurs by the metacyclic promatigostes inoculation during the female sand fly feed.

## How Parasite Promotes Survival in a Non-Friendly Environment?

### The First Step: Infection

Infection begins when a female sand fly interacts with the skin host and injects metacyclic promastigotes into the dermis. The proportion of metacyclic promastigotes delivered by the female sand fly into the skin is responsible for the success of the infection (Giraud et al., 2019). Flies infected for more days contain more parasite burden and are able to deliver higher numbers of parasites into the skin and their infective dose is enriched by non-metacyclic promastigotes (low-quality dose) (Giraud et al., 2019). This fact influences the pathology caused by *Leishmania mexicana* leading to exacerbated cutaneous lesions, enriched by neutrophils, and associated with low parasite load in the skin of mammalians (Giraud et al., 2019). A high-quality dose containing low quantity of promastigotes (~100–1,000 parasites) but enriched by metacyclic promastigotes generates milder lesions with higher parasite load and is more effective in transmitting to other sand flies, showing the influence of parasite-vector relationship on the skin inflammatory response and on the final parasite load in the mammalian host (Giraud et al., 2019). Moreover, the traumatic changes caused by the contact of proboscides with skin and the disruption of the epidermis layers associated with the sand fly saliva induce the endothelial activation and neutrophil infiltration (Peters et al., 2008; Peters and Sacks, 2009). The inflammatory infiltrate generated creates a toxic environment from which promastigotes have to escape to survive. For this, promastigotes have to escape from extracellular toxic environment, enter the host cells, and convert into amastigote form. In this context, components in the sand fly saliva may help promastigotes in this issue. For example, saliva contains endonuclease, which digests Neutrophil Extracellular Traps (NETs) and inhibits blood coagulation, possibly allowing the local spread of promastigotes (Chagas et al., 2014).

Resident macrophages together with infiltrated neutrophils are the first cells to interact with *Leishmania* parasite upon infection. Neutrophils may exert a variety of effector mechanisms, such as phagocytosis, enzymes, and antimicrobial proteins release and NET formation (Kennedy and De Leo, 2009). *Leishmania amazonensis* promastigotes induce NET formation (Guimarães-Costa et al., 2009) and NETs were also demonstrated in active and chronic lesions of Tegumentary Leishmaniasis (TL) caused by *Leishmania braziliensis*, showing that even amastigotes may induce NET formation (Guimarães-Costa et al., 2009; Morgado et al., 2015). In *in vitro* system, NETs exerted restraint and toxic effects for *L. amazonensis* promastigotes (Guimarães-Costa et al., 2009), which could be reverted by the expression of *Leishmania*- 3'Nucleotidase/Nuclease activity digesting NETs and escaping killing by released NETs (Guimarães-Costa et al., 2014). In patients infected with *L. braziliensis*, ecto-nucleotidase activity is correlated with clinical manifestations: isolates from mucosal lesions presented higher ecto-nucleotidase activity than skin lesions (Leite et al., 2012). Furthermore, higher ecto-nucleotidase activity is also correlated with higher parasite load, and modulation of immune response by inhibiting dendritic cell activation and NO production (Leite et al., 2012). The interaction between NETs and macrophages favor the survival and persistence of parasites since it stimulates the M2 profile of macrophages, which are susceptible to *L. amazonensis* infection but unable to kill the parasite (Guimarães-Costa et al., 2017). Recently, *L. panamensis* resistant strains to miltefosine and meglumine antimoniate were described (Regli et al., 2018). These strains were able to modulate neutrophils stimulating the release of NETs and ROS production and survived better within neutrophils than the drug-susceptible strains (Regli et al., 2018). Altogether, these published data suggest that the induction of NET formation may lead to escape from extracellular effector immune mechanisms, facilitating the access of a permissive cell to parasite infection and persistence.

Still in the extracellular milieu, promastigotes have to survive from the effect of complement molecules. For this, LPG present in the parasite surface interferes with the complement cascade (Hermoso et al., 1991) and with the insertion of membrane attack complex (Puentes et al., 1990). In addition, the metallopeptidase GP63 cleaves the C3b molecule (Brittingham et al., 1995).

To enter host cells, *Leishmania* spp. uses manose-fucose (Akilov et al., 2007), Fc (Kima et al., 2000), fibronectin (Brittingham et al., 1999), and Toll-like receptors (Kropf et al., 2004), as well as CR1 and CR3 complement receptors (Blackwell et al., 1985; Wenzel et al., 2012). Inside the phagolysossoma, *Leishmania* spp have to escape from the acid environment, the action of enzymes and the microbicidal effects from oxygen and nitrogen radicals. In *L. donovani* experimental infection, the inhibition of phagolysosomes biogenesis was observed (Matheoud et al., 2013). As an effect of the metallopeptidase GP63 that mediates the cleavage of SNAREs, *L. donovani* infection prevented NADPH oxidase complex from assembling, altering the pH and phagosome degradative properties (Matheoud et al., 2013). As a result, antigen presentation via MHC-I was impaired, reducing the T cell activation (Matheoud et al., 2013).

During parasite-host macrophage interaction, *Leishmania* spp. infection may exert some impacts on signaling pathways leading to the inability of macrophages to kill intracellular parasites (Awasthi et al., 2003). For example, in experimental infection of BALB/c mice with *Leishmania major*, the CD40 signaling pathway is impaired (Awasthi et al., 2003). Thus, it results in the impairment of protein kinase C (PKCα, βI, βII, and ε) (Sudan et al., 2012) and the consequent reduction in p38MAPK phosphorylation and NOS2 expression, resulting in an impairment in killing *Leishmania* amastigotes (Awasthi et al., 2003). On the other hand, *L. major* infection enhances PKCδ, ζ, and λ isoforms, promoting ERK1/ERK2 phosphorilation, IL-10 production, and parasite growth (Sudan et al., 2012). These effects can be reverted using anisomycin, a p38MAPK activator, establishing a host-protective memory T cell response (Awasthi et al., 2003). *Leishmania donovani* infection differentially regulates small G-proteins: enhances N-Ras expression, whereas it inhibits K-Ras and H-Ras expression (Husein et al., 2018). It also increases extracellular signal–regulated kinase 1/2 phosphorylation and simultaneously decreases p38 phosphorylation, leading to the reduction of IL-12 and the increase of IL-10 expression (Husein et al., 2018). In macular PKDL patients, *L. donovani* infection decreased leucocyte rolling (L-selectin shedding) and induced up-regulation of the cellular signaling factors involved in pathogenesis (ERK1/2) as well as down regulated the signaling elements (p38 MAPK) involved in the Th1 response (Singh et al., 2018). The role of IL-10-induced immunosuppression in parasite persistence in visceral leishmaniasis and PKDL has already been described (Zijlstra, 2016; Lima et al., 2017; Bunn et al., 2018; Viana et al., 2019). *Leishmania infantum* subverts the host inflammatory response through the adenosine A2_A_ receptor by inducing CD4^+^FOXP3^+^ T cells and IL-10 expression impairing the development of Th1-type adaptive immunity and promoting the establishment of infection (Lima et al., 2017). In experimental infection with *L. donovani*, the absence of IL-10 resulted in the control of parasite replication, but also caused tissue damage and the rupture of splenic microarchitecture (Bunn et al., 2018). PKDL is characterized by an intermediate position between a Th2 and Th1 response: the Th2 response shows the presence and persistence of IL-10 in the skin that was already present during VL, while systemically the Th1 response that was induced after VL therapy persists with IFN-γ production (Zijlstra, 2016).

Although not observed in *L. major* infection (Späth et al., 2008), in *L. donovani* infection, the inhibition of nitric oxide production by the protein tyrosine phosphatase (SHP-1) in mice macrophages was demonstrated *in vitro* and *in vivo* (Forget et al., 2006). The authors demonstrated that *Leishmania major* was able to inhibit the activation of JAK2 and ERK1/2 and the transcription factors NF-kB and AP-1 since SHP-1 is a negative regulator of these transcription factors (Forget et al., 2006; Blanchette et al., 2008). In addition, infection of macrophages with *L. major* induces the expression of Monarch-1, a negative regulator of NF-kB activation (Fata et al., 2013). *Leishmania infantum* infection induces the activation of phosphatidylinositol 3-kinase/Akt and extracellular signal-regulated kinase 1/2 and promotes the cleavage of the nuclear factor-kB p65^RelA^ subunit by infected DCs (Neves et al., 2010). Therefore, *Leishmania* can inhibit microbicidal oxygen and nitrogen radicals, enabling them to escape from effector mechanisms upon phagocytosis. Some of these effects could be reversed *in vitro* using meglumine antimoniate, but nitric oxide production was only recovered using the drug associated with TNF-α (Muniz-Junqueira and de Paula-Coelho, 2008).

In human cutaneous leishmaniasis, the oxidative burst of monocytes is higher than that from healthy donors (Carneiro et al., 2016). However, NO alone is not sufficient to control the parasite load (Carneiro et al., 2016). This capacity to resist or interfere with NO production by macrophages depends on the *Leishmania* species involved (Table 1; Campos et al., 2008). Therein, *L. braziliensis* isolated from Mucosal Leishmaniasis patients showed the highest infection index and the lowest NO production *in vitro* by macrophages when compared to the infection by the other species of subgenus *Viannia* (Campos et al., 2008). In contrast, *L. naiffi*, which generally causes a benign and autoresolutive infection in humans, showed the lowest infection index and the highest NO production (Campos et al., 2008). In another study, promastigotes isolated from patients with mucosal leishmaniasis express more thiol-specific antioxidant protein, and were more resistant to nitric oxide and H_2_O_2_ than strains obtained from localized cutaneous leishmaniasis (Ávila et al., 2018). *Leishmania amazonensis*, which is capable of inducing severe diffuse cutaneous form cursing with high parasite load and inactivation of macrophages, also developed strategies to escape from oxidative effects from phagocytes. For example, *L. amazonensis* activates the NF-kB repressor complex p50/p50 that negatively regulates NOS2 expression interfering with NO production (Calegari-Silva et al., 2009). *Leishmania donovani* produces ornithine decarboxylase, which plays a role in the synthesis of tripanothione, the major reduced thiol responsible for the modulation of the immune response and pathogenesis in visceral leishmaniasis (Yadav et al., 2015). In this last study, the effects of a recombinant ornithine decarboxylase from *L. donovani* (r-LdODC) on the immune response of peripheral blood mononuclear cells of patients affected by visceral leishmaniasis were observed (Yadav et al., 2015). The r-LdODC induced the production of IL-10 from CD4 T cells and the reduced IL-12 and NO production (Yadav et al., 2015). The induction of proinflammatory mediators such as TNF-α and IL-6 through the transcription factor IRF-5 by *L. donovani* has also been demonstrated (Hammami et al., 2015). IRF-5 also limits CD8 T cell expansion and inhibits IL-12 expression, favoring the establishment of persistent infection (Hammami et al., 2015).

**Table 1:** Main evasion mechanisms already described for *Leishmania* species.

***Leishmania* species**	**Escape mechanisms mainly described**	**Reference**
*L. major*	Impairment in CD40 signaling. Impairment of protein kinase C (PKCα, βI, βII and ε) and enhancement of PKCδ, ζ and λ isoforms, promoting ERK1/ERK2 phosphorilation, IL-10 production and parasite growth. Suppression of IL-2Rα expression by peripheral human T lymphocytes. Induction of Monarch-1 expression by macrophages, a negative regulator of NF-kB activation. Hijack of host cell autophagy machinery to reduce T-cell proliferation. Induction of apoptosis of lymphocytes by the downregulation of Bcl-2 and over-expression of p53 and caspase-3.	Awasthi et al., 2003 Sudan et al., 2012 Khodadadi et al., 2013 Fata et al., 2013 Crauwels et al., 2015 Moshrefi et al., 2017
*L. donovani*	Inhibition of nitric oxide production and the activation of JAK2 and ERK1/2 and the transcription factors NF-kB and AP-1 by the protein tyrosine phosphatase (SHP-1) in macrophages. Different regulation of small G-proteins, enhancing N-Ras expression, and inhibiting K-Ras and H-Ras expression. Induction of PD-1 and CTLA-4 expression by lymphocytes. Induction of PD-L2 expression by macrophages. Inhibition of phagolysosomes biogenesis, NADPH oxidase complex assembling, altering the pH and phagosome degradative properties, and inhibiting antigen presentation via MHC-I. Induction of the transcription factor IRF-5 that limits CD8 T cells expansion and inhibits IL-12 expression.	Forget et al., 2006; Blanchette et al., 2008 Husein et al., 2018 Murphy et al., 1998 Medina-Colorado et al., 2017 Matheoud et al., 2013 Hammami et al., 2015
*L. braziliensis*	Reduction of NO production and high infection index *in vitro*. Decrease of BLT1 receptor, which recognizes the lipid leukotriene B4 (LTB4). Increase of superoxide dismutase 1 (SOD1) by macrophages. Ecto-nucleotidase activity.	Campos et al., 2008 Morato et al., 2014 Khouri et al., 2014 Leite et al., 2012
*L. amazonensis*	Phosphatidylserine exposition. Activation of NF-kB repressor complex p50/p50 that negatively regulates NOS2 expression interfering with NO production. Induction of NETs and stimulation of M2 profile. Increase of superoxide dismutase 1 (SOD1) by macrophages. Downregulation of macrophage iNOS expression via Histone Deacetylase 1 (HDAC1).	Wanderley et al., 2009 Calegari-Silva et al., 2009 Guimarães-Costa et al., 2017 Khouri et al., 2014 Calegari-Silva et al., 2018
*L. infantum*	Use of apoptosis from host cell as a mean to survive and replicate. The mechanism should be clarified. Expression of 3'Nucleotidase/Nuclease activity. Manipulation of AMPK activation. Production of the enzyme ornithine descarboxylase, which plays a role in the synthesis of tripanothione and the modulation of the immune response and pathogenesis. Lower expression of TLR2, TLR9, HLA-DR and TNF-α, resulting in less control of parasite load. Induction of MHC Class IIhigh DC to polarize CD4+ T Cells toward a nonprotective T-bet+ IFN-g+IL-10+ phenotype.	Moreira et al., 2013 Guimarães-Costa et al., 2014 Moreira et al., 2015 Yadav et al., 2015 Viana et al., 2018 Resende et al., 2013
*L. guyanensis*	Induction of the up-regulation of the A20 protein, avoiding the inflammasome pathways.	Hartley et al., 2018

*Leishmania* spp can also interfere with immune cell-cell communication, inhibiting an efficient immune response. For example, *L. major* can suppress IL-2Rα expression by peripheral human T lymphocytes (Khodadadi et al., 2013). This suppression impacts the proliferation of T cells, stimulation of NK cells, and IFN-γ production, thus resulting in a failure of immune response. In this sense, *L. infantum* may induce MHC Class II^high^ DC to polarize CD4^+^ T Cells toward a non-protective T-bet^+^ IFN-g^+^IL-10^+^ phenotype, which is associated with chronicity and prolonged parasite persistence (Resende et al., 2013). Differences in the capability to modulate the effector response from monocytes by both species have also been demonstrated (Viana et al., 2018). *Leishmania infantum* infections lead to lower expression of TLR2, TLR9, HLA-DR and TNF-α, resulting in less control of parasite load, which may reflect upon the distinct clinical course observed in patients presenting visceral and tegumentary leishmaniasis (Viana et al., 2018).

Once the parasite converts into intracellular amastigotes, the next step is dissemination. A study comparing *L. major*—promastigotes and amastigotes infection of macrophages—showed that while infection with promastigotes induced inflammatory mediators, such as TNF-α and chemokines, amastigote infection was silent resulting in increased parasite load (Wenzel et al., 2012).

### The Infection Continuity: A Question of Response Subversion to Promote *Leishmania* spp Survival

Sand fly saliva is composed of multiple components with a variety of functions and is able to immunomodulate macrophages (Lerner et al., 1991; Morris et al., 2001; Oliveira et al., 2008). Maxadilan, the most well-defined component from *Lutzomyia longipalpis* saliva, alone decreases TNF-α, IL-10 and increases IL-6, IL-8, and IL-12 production in LPS stimulated human macrophages (Costa et al., 2004). In addition, saliva from *Lutzomyia longipalpis* increases the anti-inflammatory mediator PGE2 and reduces the lipid leukotriene B4 (LTB4), favoring *L. infantum* infection in C57BL6 mice (Araújo-Santos et al., 2014). *Leishmania* spp. may also affect macrophage profile and functions. Infection with *L. braziliensis* leads to the decrease of BLT1 receptor, which recognizes LTB4 (Morato et al., 2014). LTB4-BLT1 recognition activates pro-inflammatory responses and stimulates the leishmanicidal activity of macrophages and neutrophils by inducing ROS secretion (Morato et al., 2014; Plagge and Laskay, 2017). Depending on the species, *Leishmania* can differently modulate macrophages. For example, infection with *L. baziliensis* promotes the infiltration of intermediate monocytes that express TNF and IL1β and enhance the inflammatory response in human patients (Passos et al., 2015; Santos et al., 2018). LPG from *L. braziliensis* was able to induce higher TNF-α, IL-6, IL-1β, and NO production by infected macrophages than LPG from *L. infantum* (Ibraim et al., 2013). In addition, *L. braziliensis* activates NF-kB, while no ERK activation by *L. infantum* LPG was demonstrated (Ibraim et al., 2013). In fact, the production of IL-1β by cutaneous leishmaniasis patients is associated with chronic inflammation and tissue damage (Novais et al., 2017). *L. amazonensis* induces HDAC1, which negatively regulates NOS2 expression contributing to the hyporeactive state of macrophages and to the replication of amastigotes (Calegari-Silva et al., 2018). *Leishmania infantum* is able to modulate host macrophage mitochondrial metabolism by hijacking the SIRT1-AMPK axis (Moreira et al., 2015). Parasites alter the macrophage metabolism toward aerobic glycolysis (Warburg effect) with a concomitant decrease of mitochondrial function, leading to the accumulation of intracellular ATP (Moreira et al., 2015). Consequently, an activation of AMPK is observed and leads to catabolic processes to restore intracellular energy and nutrients that can nourish the parasite (Moreira et al., 2015). Besides that, *Leishmania* parasites activate the PERK/eIF2α/ATF-4 pathway in cultured macrophages and infected human tissue protecting themselves from the harmful consequences of cellular stress, thus contributing to parasite survival and progression of the infection (Dias-Teixeira et al., 2017). Recently, the modulation of human macrophages by *L. infantum*—MicroRNA hsa-miR-346 demonstrated to play a role in regulating macrophage functions since several MHC- or interferon-associated genes are targets for this miRNA (Diotallevi et al., 2018).

Apoptosis is a process of cell death that maintains tissue homeostasis (Trahtemberg and Mevorach, 2017). Apoptotic cells present morphological and functional characteristics, such as chromatin condensation, pyknotic, and fragmented nuclei, apoptotic bodies formation, and phosphatidylserine (PS) exposition (Elmore, 2007; Trahtemberg and Mevorach, 2017). PS is recognized by regulatory receptors, such as TIM-3, which inhibits the inflammatory response (Kerr et al., 2018). As an escape mechanism, promastigotes may also inhibit the inflammatory process due to exposition of PS (Wanderley et al., 2009). Different from amastigotes whose entire population expresses PS, part of *L. amazonensis* promastigotes are PS^+^ and modulate inflammatory infiltrate, while PS^−^ promastigotes are able to successfully infect macrophages and survive intracellularly due to host phagocyte inactivation and reduction of NO production (Wanderley et al., 2009). Apoptotic parasites stimulate TGF-β production, silencing macrophages and favoring the survival of viable parasites (Aga et al., 2002; van Zandbergen et al., 2004). Therefore, efficient *in vivo* and *in vitro* infection occurred only when PS positive and negative promastigotes subpopulations were added simultaneously (Wanderley et al., 2009). Apoptotic-like *Leishmania* also uses host cell autophagy machinery (Crauwels et al., 2015). Therefore, T cell proliferation is inhibited favoring the overall population survival (Crauwels et al., 2015). Autophagy machinery plays a role during homeostasis by clearing apoptotic bodies in a process known as LC3-associated phagocytosis and promotes an anti-inflammatory response with production of IL-10 and TGF-β and hampering the production of proinflammatory cytokines, such as TNF-α, IL-1B, and IL-6 (Crauwels et al., 2015), generating a microenvironment favorable to *Leishmania* survival and replication.

Still at the beginning of infection, the infiltrating neutrophils rapidly respond to the infection and phagocytize the promastigotes and die generating apoptotic bodies that inhibit the response of macrophages (Afonso et al., 2008; Ribeiro-Gomes and Sacks, 2012). In established infection, apoptosis of host cells has been correlated with disease progression and increase of parasite load, suggesting the parasite can use the normal regulatory process to escape from effector mechanisms from leucocytes (Moreira et al., 2013). *Leishmania major* infection induces apoptosis of lymphocytes by the down-regulation of Bcl-2 and over-expression of p53 and caspase-3 (Moshrefi et al., 2017). However, how *Leishmania* spp may use this mechanism to evade immune response has yet to be clarified. In fact, apoptosis of inflammatory cells may occur as a result of cellular exhaustion (Liu et al., 2018). Cellular exhaustion was first described in chronic viral infection and manifested at the beginning of infection, being characterized by the expression of PD-1 receptor (Programmed Death 1) (Barber et al., 2006). PD-1 expression is induced by repeated antigenic stimulation of T and B lymphocytes (Eichbaum, 2011). The PD-ligand 1 (PD-L1) is constitutively expressed by B and T lymphocytes, macrophages and dendritic cells from spleen (Eichbaum, 2011). PD-1 activation induces apoptosis, down-regulates cellular proliferation and cytokine expression (Joshi et al., 2009). PD-1 and CTLA-4 expression by CD8 cells from peripheral blood and spleen were demonstrated in human VL (Gautam et al., 2014) and in *L. donovani*-infected mice (Murphy et al., 1998). The exhaustion pathway blockage with anti-CTLA-4 antibody increased IFN-γ and IL-4 expression in murine spleen and liver, as well as accelerated the development of hepatic granulomatous response associated with the reduction of parasite load (Joshi et al., 2009).

Esch et al. (2013) studied dogs with VL that showed CD4^+^PD-1^+^ and CD8^+^PD-1^+^ cells in peripheral blood. These cells presented reduced proliferation index, as wells as reduced IFN-γ expression. Blockage of the exhaustion pathway *in vitro* recovered the T cell effector functions, such as proliferation index and oxidative radical production. As a result, the parasite load was partially controlled by monocytes. Exhaustion of macrophages has also been demonstrated in *L. donovani* infection (Medina-Colorado et al., 2017). The blockage of PD-L2 was able to induce a reduction in arginase-1 and to control the parasite load; however, no alterations in IFN-γ nor NOS2 were demonstrated (Medina-Colorado et al., 2017). Despite the Th1 response induced and the IFN-γ expression detected during leishmaniasis, amastigotes are able to survive probably by using normal regulatory mechanisms developed by the immune system that prevents tissue damage and maintains the homeostasis. In this context (Kong et al., 2017), observed that IFN-γ enhanced *L. donovani* growth and induced the counter-regulatory molecules STAT3, IL-10, Arg1, Ido1, and Irg1 in splenic macrophages, which suggests that splenic macrophages in VL are conditioned to respond to macrophage activation signals with a counter-regulatory response, which is ineffective and even disease-promoting (Kong et al., 2017). M2 macrophages were also observed during *Leishmania* spp infection (Moreira et al., 2016; Hu et al., 2018; Kumar et al., 2018; Lee et al., 2018). The M2 profile has been described as a cell type that participates in the processes of cellularity reduction in the inflammatory infiltrate, tissue remodeling, and healing (Tomiotto-Pellissier et al., 2018). In this regard, it produces IL-10, TGF-b, and endothelium growth factors make a cell unable to express NOS2 and kill intracellular amastigotes, favoring *Leishmania* replication and persistence.

Recently, the capacity of *Leishmania* spp to modify its genome constitution in order to better adapt to different environments has been demonstrated for *L. donovani, L. major*, and *L. tropica* complexes (Prieto Barja et al., 2017; Bussotti et al., 2018). Aneuploidy has been described for *Leishmania* parasites and this phenomenon generates karyotypic fluctuations that are correlated with phenotypic variations, impacting the proliferative, and infectivity capacities (Prieto Barja et al., 2017). *Leishmania* clones containing beneficial haplotypes selected by the environment give rise to specific copy number variation profiles that could represent a fitness gain to new hostile environments (Bussotti et al., 2018). Therefore, *Leishmania* is able to change its chromosome and gene copy number to adapt to environmental changes (Prieto Barja et al., 2017; Bussotti et al., 2018). In this sense, the microenvironment generated by immune cells from the mammalian host or by anti-*Leishmania* drugs may constitute a bottle neck for the selection of better adapted clones, enabling the persistence of parasites. For example, the allopurinol-resistance of *L. infatum* strain was associated with chromosome and gene copy number variations (Yasur-Landau et al., 2018). The gene encoding the s-adenosylmethionine synthetase (METK) showed diminished copy numbers in allopurinol-resistance strains (Yasur-Landau et al., 2018). The deletion of the gene LinJ.36.2510 that encodes 24-sterol methyltransferase was associated to amphotericin B-resistance (Rastrojo et al., 2018). And whole genomic sequencing of *L. mexicana* strain showed an association between anphotericin B resistance and mutation of the sterol biosynthesis gene (Pountain et al., 2019). Moreover, a comparative proteomic analysis revealed the role of the iron superoxide dismutase in miltefosine resistance of *L. donovani* strains (Veronica et al., 2019). In another study, genomic analysis of *L. infantum* strains evidenced an association between deletion or mutations in LiMT and LiRos3 genes and miltefosine-resistance (Mondelaers et al., 2016). Finally, the increase of thiol levels was associated to antimony-resistance indicating that the redox metabolism has a role in the antimony-resistance of new world VL isolates (Magalhães et al., 2018).

Once the parasite is able to successfully establish growth and survival in the cells from mammalian host, clinical disease can be detected. However, a certain number of infected individuals are able to control parasite replication as well as the immune response, becoming permissive to parasite persistence. In this case they are considered infected but not sick. In this context, patients with subclinical infection with *L. braziliensis* may present more CD8^+^IFN-γ^+^ cells and less Cytotoxic CD8^+^ T cells than patients manifesting cutaneous leishmaniasis (Cardoso et al., 2015). However, even these subclinical patients maintain latent parasites that may reactivate in case of immunosuppression (Conceição-Silva et al., 2018). How do parasites persist in mammalian tissues? How does mammalian host contain parasite replication? Answering these questions is crucial to understand the role of both sides to survive without serious damage. Consequently, this knowledge may improve the design of new methods for diagnosis, the development of new drugs, as well as the way to construct a vaccine.

## Is There an End in *Leishmania* spp Infection? an Update

Recently, some factors enrolled in parasite persistence were reviewed (Conceição-Silva et al., 2018). In this connection, the *Leishmania* spread and persistence in different sites have been well-characterized by different authors. Thus, it was already described that in active cutaneous leishmaniasis patients, the parasite can be present in extralesional sites (Romero et al., 2010; Canário et al., 2019). In this subject, Canário et al. (2019) showed that positivity in healthy mucosa was high in patients with more severe cutaneous lesions and those who needed to be treated longer. However, we still do not know whether parasite burden can influence parasite persistence. Evidences of parasite persistence were not restricted to cutaneous leishmaniasis. In visceral leishmaniasis, parasites may persist after clinical cure of primary manifestations leading to PKDL (Ganguly et al., 2010; Singh et al., 2012; Zijlstra, 2016) or to visceral leishmaniasis reactivation in HIV patients (Freitas-Junior et al., 2012; Távora et al., 2015; Henn et al., 2018). In addition, the presence of some differences in evasion mechanisms according to *Leishmania* infection produced by different species (de Freitas et al., 2016, among others) has been shown.

**Figure 1:**
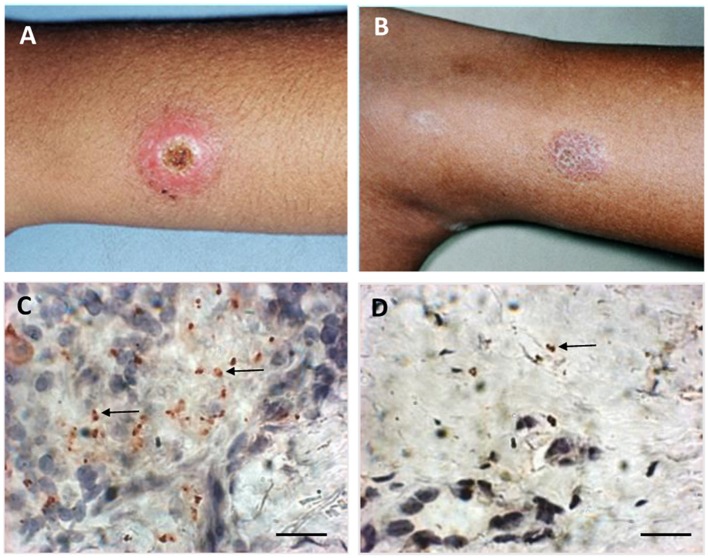
Macroscopic and microscopic aspects of active lesion and scar of American Tegumentary Leishmaniasis. **(A)** Active lesion—single ulcer with elevated borders and granulomatous aspect in center. **(B)** Scar—atrophic scar after successful treatment. **(C)** Several amastigotes detected by immunohistochemistry (arrows) involved by a granulomatous reaction in an active lesion. **(D)** Two amastigotes (arrow) detected by immunohistochemistry near a discrete inflammatory reaction characterized by cell niches in a scar. Magnification bar = 10 μm.

Direct or indirect evidences of parasite persistence after healing have been demonstrated since the 1990's (reviewed by Conceição-Silva et al., 2018) (Figure 1). Thus, the importance of the mouse genetic background in the development of disease or parasite persistence has been explored by different research groups (reviewed by Launois et al., 1997; Fowell and Locksley, 1999; Conceição-Silva et al., 2018). In addition, *Leishmania* profile has also been implicated in different degrees of infection and healing (De Luca and Macedo, 2016). It was already discussed the *Leishmania* capacity to modify its genome in order to increase the survival in hostile environment (Prieto Barja et al., 2017; Bussotti et al., 2018, see above). This phenomenon would help parasite to survive either during active disease or healing phase.

One of the first pieces of information about how parasite could persist after leishmaniasis healing showed that parasites could subvert the production of reactive nitrogen and oxygen intermediates and modulate the cytokine production, such as interleukin 12, by the host cells (Bogdan et al., 1996; Stenger et al., 1996; Bogdan and Röllinghoff, 1998). These functions have been detected both *in vitro* and *in vivo* using mouse model of *L. major* infection. Later on, in animal model, it was shown that, in addition to the parasites inside macrophages, 40% of the persistent parasites were associated with fibroblasts with reduced expression of NOS2, pointing the importance of these cells to promote parasite survival (Bogdan et al., 2000). It was also demonstrated that GP63 metalloprotease from parasite can inhibit key microbicidal activity by altering antigen presentation and important metabolic and signaling pathways subverting macrophage function in controlling parasite growth (reviewed by Duque and Descoteaux, 2015; Soulat and Bogdan, 2017). In addition, it was also shown that *Leishmania* parasites harboring *Leishmania* RNA virus 1 (LRV1) can promote macrophage survival via LRV1 recognition through TLR-3. The authors showed in an *in vitro* model the possibility of LRV1 interference leading therefore to an increased infection in macrophages (Eren et al., 2016). Hence, the concomitant presence of parasitized macrophages and fibroblasts could produce a balance between death and survival, which would allow the control of parasite growth via continuous immunological stimulus and at the same time would allow a continuous presence of live parasites. In this sense, the persistence parasite at the primary infection site and as a result the continuous antigenic stimulus that leads to long-lasting immunity to reinfection was discussed previously (Okwor and Uzonna, 2008). Nowadays, pieces of evidence of parasite persistence in leishmaniasis are getting more robust. However, we still do not know a real percentage of patients that maintain a tissue parasitism and exactly how the host keeps the parasite under control. Hence, detailed information of how it does occur and the implication of this persistence in the protection to reinfection are still under construction. As tissue parasite distribution seems to be heterogeneous, and different methodological approaches can influence the positivity, one cannot affirm that negative cases reached a sterile healing. In this context, Morgado et al. (2010) showed by immunohistochemistry a gradual reduction of inflammatory reaction after clinical cure, which could be observed even in 3-year-old scars (Morgado et al., 2010). The maintenance of inflammatory profile restrict to niches of cells was associated with the evidence of parasites in 2 scars from evaluated patients (Conceição-Silva et al., 2010; Morgado et al., 2010).

PCR detection of parasites has been widely used as a method to identify *Leishmania* spp in different tissues, but the primer design is considered crucial to detect parasites, since it has been shown that the use of different primers can produce different percentage of positivity (Romero et al., 2010). Nevertheless, parasites have been detected by PCR in different tissues upon infection, even in the absence of clinical disease. Authors have reported the presence of viable parasites in nasal, oral and conjunctival mucosa as well as in blood mononuclear cells, tonsils and normal skin in asymptomatic individuals and also in patients with isolated cutaneous lesions, demonstrating parasite dissemination without evidences of disease (Martinez et al., 1992; de Oliveira Camera et al., 2006; Vergel et al., 2006; Figueroa et al., 2009; Romero et al., 2010; Rosales-Chilama et al., 2015, among others). Figueroa et al. (2009) showed the presence of *Leishmania* spp in 81% of nasal tissues of patients with active cutaneous leishmaniasis without clinical signs of nasal lesion. In addition, Martinéz-Valencia et al. (2017) detected by PCR-Southern blot a percentage of positivity in 30% of the healed patients evaluated 13 weeks after treatment initiation. Rosales-Chilama et al. (2015) detected parasites in 40% of healthy individuals living in endemic areas and presenting positivity to Montenegro skin test (MST+). These authors were able to demonstrate parasite viability in 59% of the MST+ individuals. Interestingly, they were also able to detect parasites in few negative MST volunteers. Taken together, these results confirm the dissemination of *Leishmania* parasites in different host tissues during the active disease. They also confirm the persistence of parasites in humans after healing, and most importantly, even in healthy individuals without clinical signs of past active or healed tegumentary leishmaniasis.

Protozoan persistence seems to be very frequent in infected individuals, and the onset of an immunosuppression can lead to a recrudescent disease, even in those cases in which the primo infection was silent (discussed in Conceição-Silva et al., 2018). In leishmaniasis, the distribution of the parasite in the tissues from mammalian host appears to occur very early during the infection. In this sense, these results presented above confirm that, at least in leishmaniasis caused by the subgenus *Viannia*, parasites are precociously located in the mucosae of the upper airways and digestive tracts. However, it has not yet been proven whether this fact facilitates the appearance of secondary mucosal lesions.

What are the advantages or disadvantages of parasite persistence to the host? One possibility is to protect individuals against new infection. However, it was already demonstrated that, at least in mouse model, the immune response elicited by the persistent parasite do not always avoid the possibility of a re-infection (Mandell and Beverley, 2016, among others). On the other hand, the infection does not necessarily evolve into the disease, and even if reinfection is not avoided, the persistence of the parasite may prevent the development of clinical manifestations as long as the immune system is competent. The understanding of this complex host-parasite interaction and the identification of favorable and effective immune responses capable maintaining this equilibrium could be used in vaccine development (Okwor and Uzonna, 2008; De Luca and Macedo, 2016). In addition, Mandell and Beverley (2016) discussed that persistent infection of *L. major* would work as a “natural vaccination” in a condition known as concomitant immunity. Although concomitant immunity seems to be an advantage for the host, this interaction also promotes a potential dangerous condition since if one of the actors modifies the profile (i.e., increased parasite virulence or immunosuppression) the host-parasite equilibrium can be broken leading to the development of severe disease. In this context, the authors showed in a mouse model of leishmaniasis resistance that after a secondary challenge parasites are able to colonize and persist as much as the parasites from the primary infection. However, disease is weak when compared with the primary clinic onset. According to the authors, these results indicated that this strategy would facilitate the generation of parasite diversity during the life cycle maintenance. The same authors (Mandell and Beverley, 2017), using mouse model of *Leishmania major* infection, described the presence of two parasite populations: one with normal replication rate and a second one with very low replication. In addition, they also demonstrated parasites inside NOS^+^ cells, indicating a dynamic control of parasite burden as the number of parasites keeps almost the same throughout the study. They concluded that the constant parasite replication and death are implicated in concomitant immunity and thus are beneficial to both parasite and host. It was not observed an attenuation of NO production by the increase of substrate competition. Both NOS2 and arginase utilize L-arginine as substrate and Paduch et al. (2019) demonstrated that Arginase I (Arg1)-deficient C57BL/6 mice were able to control *Leishmania* infection as much as the wild type. The absence of Arg1 did not affect parasite burden, NO production, or host cell parasitism. In wild type, they detected Arg1 in skin during active lesions but not in healed tissue. The authors also showed parasites in NOS2 negative areas enriched by myeloid cells and fibroblasts. In conclusion, the authors indicated that Arg1 is not essential to parasite control and lesion healing. On the other hand, arginase was already detected in *Leishmania* parasites, mainly in glycosomes (da Silva et al., 2012; Soares-Bezerra et al., 2013). Arginase pathway is necessary to produce polyamines, which have multiple roles in stabilizing nucleic acids and membranes, as well as regulating cell growth and differentiation. In this context, it has been demonstrated the importance of arginase activity in parasites, since replication is mainly dependent on polyamines (Badirzadeh et al., 2017). Nevertheless, it has already been demonstrated a NO production by *Leishmania* parasites (Géigel and Leon, 2003; Genestra et al., 2003a,b,c), and *Leishmania*-NO seems to have a role during host-parasite interaction (Temporal et al., 2005; Badirzadeh et al., 2017).

Recently, Holowka et al. (2016) studied the influence of *Leishmania*-encoded othologs of macrophage migration inhibitory factor in parasite survival and persistence, using KO parasites (mif^−/−^) to cytokine macrophage migration inhibitory factor (MIF) and a wild type (mif^+/+^) as control. The results showed that mice infected with mif^−/−^ parasites had three times less parasites, a decrease in antigen presenting cells activation, as well as in T cell priming and CD4 effector cells, leading to diminished inflammation signs. On the other hand, the infection promoted differences in the expression of exhaustion markers when compared with the infection produced by the mif^+/+^ *Leishmania major*. Taken the results together, the authors pointed out the influence of MIF produced by *L. major* to facilitate parasite persistence. In this aspect, Soulat and Bogdan (2017) reviewed the influence of parasites and host phosphatases on the host immune response mainly by modulating macrophage function. This group of molecules would act deactivating phagocytes' host, by inhibiting or stimulating different pathways of activation. One example, amongst others, is the reduced ROS release provoked by GP63, one of the most abundant *Leishmania* surface molecules. Other phosphatases can also act on macrophages function, allowing parasite survival (reviewed by Soulat and Bogdan, 2017).

## Final Considerations

In summary, results published until now point out an important influence of *Leishmania* species and their intrinsic diversity to promote and sustain parasite survival and persistence after infection. It could be detected in patients after clinical disease healing as well as in healthy individuals from endemic areas, without any signs of past sickness, and in tissues where no clinical signs of lesion were observed. Understanding the dynamic of parasite spread and tropism (if any), and the evasion mechanisms enrolled in persistence can indicate new steps for the design of new drugs and vaccines in order to control leishmaniasis.

## Author Contributions

FC-S and FM conceived the manuscript and wrote, reviewed, and approved the manuscript. FC-S conceived the figure. FM conceived the table.

### Conflict of Interest

The authors declare that the research was conducted in the absence of any commercial or financial relationships that could be construed as a potential conflict of interest.
